# Analysis and Calculation of Stability Coefficients of Cross-Laminated Timber Axial Compression Member

**DOI:** 10.3390/polym13234267

**Published:** 2021-12-06

**Authors:** Qi Ye, Yingchun Gong, Haiqing Ren, Cheng Guan, Guofang Wu, Xu Chen

**Affiliations:** 1 Institute of Wood Industry, Chinese Academy of Forestry, Beijing 100091, China; yeqi97@bjfu.edu.cn (Q.Y.); renhq@caf.ac.cn (H.R.); gfwu@caf.ac.cn (G.W.); 18751856915@163.com (X.C.); 2 School of Technology, Beijing Forestry University, Beijing 100083, China; cguan6@bjfu.edu.cn

**Keywords:** cross-laminated timber, stability coefficient, axial compression, timber structures, slenderness ratio

## Abstract

Cross-laminated timber (CLT) elements are becoming increasingly popular in multi-storey timber-based structures, which have long been built in many different countries. Various challenges are connected with constructions of this type. One such challenge is that of stabilizing the structure against vertical loads. However, the calculations of the stability bearing capacity of the CLT members in axial compression in the structural design remains unsolved in China. This study aims to determine the stability bearing capacity of the CLT members in axial compression and to propose the calculation method of the stability coefficient. First, the stability coefficient calculation theories in different national standards were analyzed, and then the stability bearing capacity of CLT elements with four slenderness ratios was investigated. Finally, based on the stability coefficient calculation formulae in the GB 50005-2017 standard and the regression method, the calculation method of the stability coefficient for CLT elements was proposed, and the values of the material parameters were determined. The result shows that the average deviation between fitting curve and calculated results of European and American standard is 5.43% and 3.73%, respectively, and the average deviation between the fitting curve and the actual test results was 8.15%. The stability coefficients calculation formulae could be used to predict the stability coefficients of CLT specimens with different slenderness ratios well.

## 1. Introduction

In recent years, cross-laminated timber (CLT) has been widely used as a prefabricated engineered wood product for wall panels, roof panels, and floor panels in mass timber construction and is composed of orthogonally oriented multi-layers of solid-sawn or engineered lumber glued with structural adhesive or mechanical fasteners such as dowels or nails [1,2]. As a kind of new structural material, there are many advantages for CLT. When compared with light wood-based construction, CLT constructions have better performance in fire including higher duration of fire resistance and greater ultimate load [3,4]. When compared with concrete constructions, CLT constructions have a lower thermal conductivity coefficient with the same thickness [5,6]. In addition, there are some other advantages of CLT construction including faster on-site construction times, lighter weight materials, use of a sustainable natural resource, carbon sequestration, lower embodied energy, and lower greenhouse gas emissions [7,8,9]. For these reasons, CLT elements have been successfully used in multi-storey timber-based structures.

Previous studies on CLT have mainly focused on the properties of the material itself such as bending, tensile, shear, and compressive strength [10,11,12,13,14,15,16]. Effects of thickness, wood species, lamina combinations, and so on [10,11,12] on the mechanical properties and properties of the CLT composites [13,14] were investigated. Our group also conducted some studies on CLT such as the size effect and prediction of compressive strength [15,16]. However, when using CLT in multi-storey timber-based structures, new challenges in stability bearing capacity need to be handled. Wei et al. [17] compared the axial compression properties between the CLT column and GLT (glued-laminated timber) column and the result showed that the CLT column specimens had worse stability bearing capacity but better ductility and energy absorption than GLT. Pina et al. [18] studied the effect of the number of layers, the size and location of openings, and angle between loading direction and longer edge of openings on CLT walls, but there are few formulae to calculate the stability bearing capacity of CLT. As a newly engineered wood product, the calculation of the stability bearing capacity of CLT members in axial compression in the structural design are not fully resolved. CLT stability coefficient calculation formulae from Canadian [19] and American [20] standards followed the calculation formulae of GLT, and formulae from the European [21] standard used the γ coefficient method to calculate the bending stiffness and then the stability coefficient. The structure of CLT is different from GLT, and the shear deformation of the CLT transverse layer should be considered. Therefore, this study aims to determine the stability bearing capacity of the CLT members in axial compression by experiments and to propose a calculation method of the CLT stability coefficient.

## 2. Stability Coefficient Calculation Formulae in Selected Countries

### 2.1. Stability Coefficient Formulae in Canada

Canadian wood structure product design code, CSA O86-2019 [19] uses a simplified method to calculate the stability coefficient of CLT products. The stability coefficient calculation followed the formulae of GLT, but some parameters such as the calculation of slenderness ratio were adjusted. The calculation formulae are shown in Equations (1)–(7).

(1) $$\varphi ={\left[1.0+\frac{F_{C}K_{ZC}C_{C}{}^{3}}{35E_{05}\left(K_{SE}K_{T}\right)}\right]}^{-1}$$

(2) $$F_{C}=f_{c}\left(K_{D}K_{H}K_{SC}K_{T}\right)$$

(3) $$K_{ZC}=6.3{\left(\sqrt{12}r_{eff}l\right)}^{-0.13}\leq 1.3$$

(4) $$C_{C}=L_{e}/\sqrt{12}r_{eff}$$

(5) $$L_{e}=K_{e}l$$

(6) $$r_{eff}=\sqrt{I_{eff}/A_{eff}}$$

(7) $$I_{eff}=\sum_{i=1}^{n}\left(I_{i}+A_{i}a_{i}{}^{2}\right)$$

where φ  is the stability coefficient under the axial compression of CLT;$\;f_{c}$ is the standard strength in compression parallel to the grain of the laminae; $F_{c}$ is the design value of compressive strength; $K_{D},K_{H},K_{SC},K_{T}$ are the load influence coefficient, system influence coefficient, environmental coefficient, and protective treatment coefficient, which are taken as 0.65, 1.0, 1.0, 1.0, respectively; $K_{ZC}$ is the size-adjustment coefficient of compression resistance; $C_{c}$ is the slenderness ratio of the specimen; $E_{05}$ is the 5% quantile of elastic modulus of laminae, which was taken as 0.82 times the standard value of the modulus of elasticity $E_{50}$ (also regarded as the mean value); $K_{SE}$ is the environmental coefficient of elastic modulus, taken as 1.0; $L_{e}$ is the calculated length of the CLT component; $K_{e}$ is the calculated length adjustment coefficient, taken as 1.0 for simple support at both ends; l is the length of the laminate; $r_{eff}$ is the effective turning radius of CLT section; $I_{eff}$ is the effective moment of inertia of CLT; $A_{eff}$ is the effective cross-sectional area of CLT; $I_{i}$ is the moment of inertia of the *i*-th laminate; $A_{i}$ is the cross-sectional area of the i-th laminate; and $a_{i}$ is the distance between the centroid of the *i*-th laminate and the intermediate laminate.

### 2.2. Stability Coefficient Formulae in Europe

The European code for the design of timber structure products, Eurocode 5: Design of timber structures [21] does not have a separate description of the stability of CLT, so the relevant content of GLT is used to calculate the effective moment of inertia and the stability coefficient of CLT by the γ coefficient method. The formulae in the European standard are shown in Equations (8)–(15).

(8) $$\varphi =\frac{1}{k+\sqrt{k^{2}-\lambda_{rel}{}^{2}}}$$

(9) $$k=0.5\left[1+\beta_{c}\left(\lambda_{rel}-0.3\right)+\lambda_{rel}{}^{2}\right]$$

(10) $$\lambda_{rel}=\frac{\lambda }{\pi }\sqrt{\frac{f_{c}}{E_{05}}}$$

(11) $$\lambda =L_{e}\sqrt{\frac{A_{eff}}{I_{eff}}}$$

(12) $$L_{e}=K_{e}l$$

(13) $$I_{eff}=\frac{{\left(EI\right)}_{eff}}{E_{50}}$$

(14) $${\left(EI\right)}_{eff}=\sum_{i=1}^{n}\left(E_{50,i}I_{i}+\gamma_{i}E_{50,i}A_{i}a_{i}{}^{2}\right)$$

(15) $$\gamma_{i}=\frac{1}{1+\frac{\pi^{2}E_{50,i}h_{i}}{L_{e}{}^{2}}\cdot \frac{h_{0}}{G_{90,0}}}$$

where k is instability coefficient; $\lambda_{rel}$ is the relative slenderness ratio of CLT; $\beta_{c}$ is taken as 0.1 (as same as glued laminated timber); λ is the slenderness ratio of CLT; $E_{50,i}$ is the standard value of the modulus of elasticity of the *i*-th laminate; $\gamma_{i}$ is the connection effect coefficient of the *i*-th laminate; $h_{i}$ is the thickness of the *i*-th laminate; $h_{0}$ is the thickness of the intermediate laminate; $G_{90,0}$ is the rolling shear modulus of the intermediate laminate, in the empirical formula, the rolling shear modulus of the laminate is taken as 1/10 of the shear modulus, the shear modulus of the laminate is taken to be 1/16 of the modulus of elasticity; the rest of the notations have the same meaning as before.

### 2.3. Stability Coefficient Formulae in the USA

American wood structure design code, NDS-2018 [20] adopts the shear analogy method for the calculation of the stability coefficient, following the formulae of calculating the stability coefficient of the GLT, but introducing the apparent bending stiffness in the calculation of the parameters to reflect the transverse shear effect of the middle layer, as shown in Equations (16)–(21).

(16) $$\varphi =\frac{1+\left(F_{cE}/F_{c}\right)}{2c}-\sqrt{{\left[\frac{1+\left(F_{cE}/F_{c}\right)}{2c}\right]}^{2}-\frac{F_{cE}/F_{c}}{c}}$$

(17) $$F_{cE}=\frac{\pi^{2}{\left(EI\right)}_{app-min}}{A_{eff}L_{e}{}^{2}}$$

(18) $${\left(EI\right)}_{app-min}=\frac{0.5184{\left(EI\right)}_{eff}}{1+K_{s}\frac{{\left(EI\right)}_{eff}}{{\left(GA\right)}_{eff}l^{2}}}$$

(19) $$L_{e}=K_{e}l$$

(20) $${\left(EI\right)}_{eff}=\sum_{i=1}^{n}\left(E_{i}I_{i}+E_{i}A_{i}a_{i}{}^{2}\right)$$

(21) $${\left(GA\right)}_{eff}=\frac{a^{2}}{\frac{h_{1}}{2G_{1}b}+\sum_{i=2}^{n-1}\frac{h_{i}}{G_{i}b}+\frac{h_{n}}{2G_{n}b}}$$

where $F_{cE}$ is the critical buckling design value for compression members; c is the material-related parameter, taken as 0.9 (the same as for glued laminated timber); ${\left(EI\right)}_{app-min}$ is the reference apparent bending stiffness of CLT for panel buckling stability calculation; ${\left(EI\right)}_{eff}$ is the effective bending stiffness of the CLT section; $K_{s}$ is the shear deformation adjustment coefficient, taken as 11.8; ${\left(GA\right)}_{eff}$ is the effective shear stiffness of the CLT section; $E_{i}$ is the modulus of elasticity of the *i*-th laminate, the elastic modulus of the laminate perpendicular to the grain is about 1/30 of the elastic modulus along the grain; $G_{i}$ is the shear modulus of the *i*-th laminate, with the longitudinal laminate taking the shear modulus and the transverse laminate taking the rolling shear modulus; and a is the centroid distance of the outermost two laminates.

### 2.4. Modification Based on Formulae in Selected Countries

The stability coefficient formulae of CLT in axial compression are stipulated based on the existing calculation methods in the timber structure design codes and related technical manuals of other countries. The stability coefficient is related to strength and the bending stiffness of the member. The values of compressive strength of CLT with different grades can be obtained from a certain standard, but the calculation methods of the effective bending stiffness are different. The Canadian standard uses the simplified method to calculate the effective bending stiffness, and the American standard uses the shear analogy method, while the European standard uses the γ coefficient.

**Figure 1:**
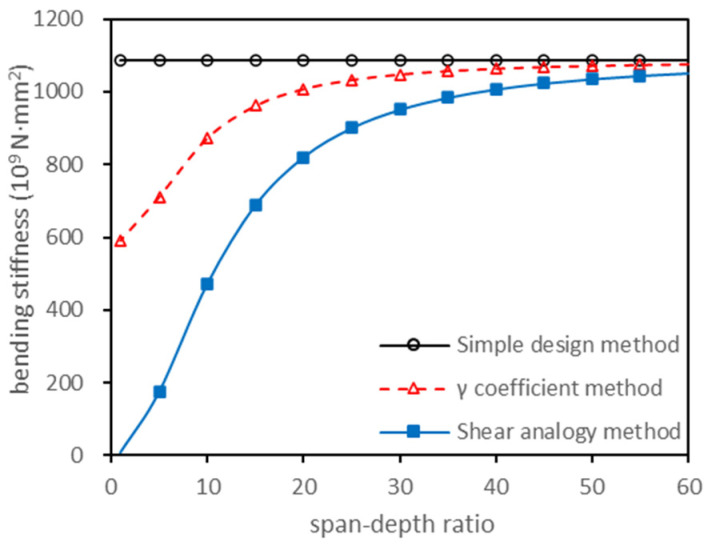
Effective bending stiffness curves with different methods.

Based on the CLT laminate parameters of the E1 grade set in the North American standard: ANSI/APA PRG 320-2019 [22], setting the width of the laminate as 1000 mm, three different methods were used to calculate the effective bending stiffness of the CLT component. The results are shown in Figure 1. It found that there exists a significant difference between the values by these three different methods when the slenderness ratio is small. However, with the increase in slenderness ratio, the values of effective bending stiffness using a different method approached the same number. The Chinese standard GB50005-2017, Standard for design of timber structure [23], stipulates that the calculation of CLT effective bending stiffness should have a simple design method, but this method ignored the interlaminar shear impact. The shear analogy method takes the shear deformation of each layer into account, but the method may only be used for relatively high slenderness ratios. The γ coefficient method originated from joggle beam theory, where the transverse laminates are used as the connecting parts between the longitudinal laminates, which takes the shear deformation of the longitudinal and transverse layers into account. It satisfies the action mechanism of CLT under axial load. Therefore, the γ coefficient method was selected to calculate the effective bending stiffness of CLT.

The different process mode of the effect on the loading duration and the treatment methods of the resistance partial coefficient can also produce different calculation results of the stability coefficient. The differences between countries are shown in Table 1.

**Figure 2:**
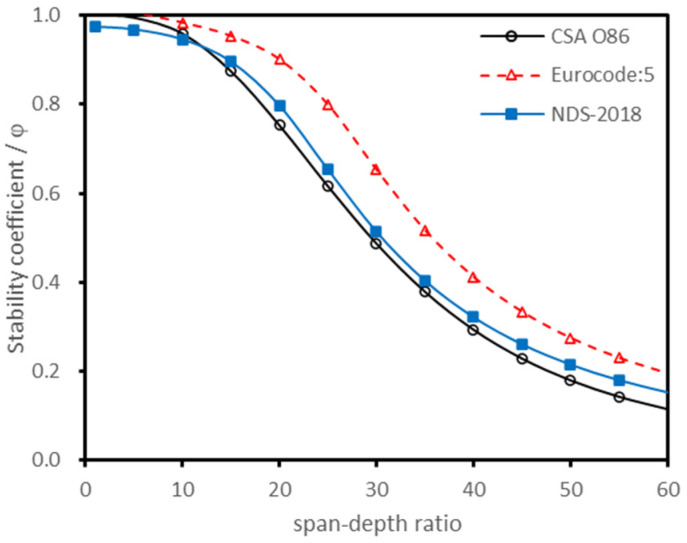
Stability coefficient curves of different countries.

**Figure 3:**
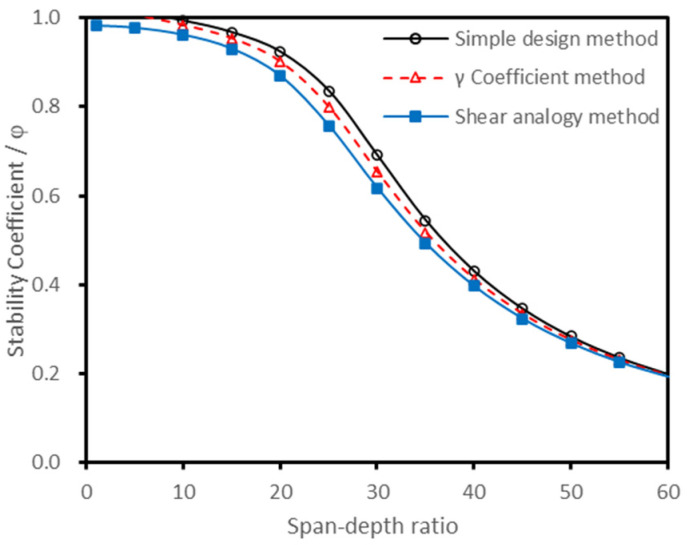
Stability coefficient curves of different calculation methods under the same treatment.

Based on the parameters of the E1 grade laminates, the stability coefficient of CLT was calculated according to the methods of different national standards, while the results are shown in Figure 2. In addition, the effective bending stiffness of CLT was calculated by the simple design method, γ coefficient method, and shear analogy method, and then the effective bending stiffness was brought into the European formulae (Equations (8)–(15)) to calculate the stability coefficient, the results of which are shown in Figure 3.

Figure 2 shows that there were significant differences between the stability coefficient curves of different countries. In contrast to Figure 2 and Figure 3, it was found that when the treatment method of the loading duration and the resistance partial coefficient were unified, the calculated stability coefficients showed little difference, even if the effective bending stiffness calculation method was different.

Zhu et al. [24] proposed a method for calculating the stability coefficient of components that was used in timbers and logs, imported dimension lumber, and glued laminated timber. This method determines the material coefficient values of different engineering wood products through regression analysis and the research results were adopted by GB 50005-2017. However, the calculation method of the stability coefficient for CLT products has not been given in the standard. Because CLTs are constructed differently to GLT and logs, the calculation method of the CLT stability coefficient cannot simply adopt the calculation method of the laminate and glued laminated timber.

Based on the compression stability coefficient calculation method of wood components in Chinese standard GB 50005-2017, Standard for design of timber structure and theoretical regression analysis, the stability coefficient formulae for CLT in this paper were simply proposed and the values of the material coefficients in the formulae were obtained. Then, the stability bearing capacity and strength bearing capacity of CLT members were tested through experiments to verify the correctness of the formulae and regression results.

## 3. Establishment of Stability Coefficient Calculation Formulae in China

### 3.1. Slenderness Ratio Formula

Compared with GLT and lumber, the shear deformation of the CLT transverse layer was relatively larger, and its influence on the stability coefficient should be considered. CLT can be regarded as a split limb, with the longitudinal lamina as the column limb and the transverse lamina as the connection between the component limbs. The influence of shear deformation on the stability coefficient of the transverse laminate can be considered by the slenderness ratio conversion theory. Therefore, according to the bar buckling theory, the formula proposed by Dr. Wu was introduced to calculate the slenderness ratio as Equation (22).

(22) $$\lambda_{eff}=\sqrt{\lambda +\frac{n\pi^{2}E_{50}A_{eff}}{{\left(GA\right)}_{eff}}}$$

where $\lambda_{eff}$ is the slenderness ratio after taking the shearing effect into account; *n* is 1.2 when the cross section is a rectangle.

### 3.2. Stability Coefficient Formulae

Based on the stability coefficient calculation formulae in GB 50005-2017, the shear deformation of the longitudinal and transverse layers was taken into account and the slenderness ratio was used; the calculation formulae are shown in Equations (23)–(26).

(23) $$\varphi =\frac{a_{c}\pi^{2}E_{05}}{\lambda_{eff}{}^{2}f_{c}}\left(\lambda >\lambda_{p}\right)$$

(24) $$\varphi ={\left(1+\frac{f_{c}\lambda_{eff}{}^{2}}{b_{c}\pi^{2}E_{05}}\right)}^{-1}\left(\lambda <\lambda_{p}\right)$$

(25) $$\lambda_{p}=c_{c}\sqrt{\frac{E_{05}}{f_{c}}}$$

(26) $$c_{c}=\pi \sqrt{\frac{a_{c}b_{c}}{b_{c}-a_{c}}}$$

where $\lambda_{p}$ is the critical slenderness ratio of the specimens; $I_{eff}$ should be obtained by the γ coefficient method; $a_{c},\;b_{c},\;c_{c}$ are the correlation coefficients of CLT components. The correlation coefficients of logs, dimension lumber, and glued laminate have been determined in the Chinese standard, but there is still no final conclusion about the stability coefficient of CLT components.

### 3.3. Stability Coefficient Parameters

The correlation coefficients of $a_{c},\;b_{c},\;c_{c}$ were obtained by the least square method. In the process of fitting, the corresponding relationship between slenderness ratio and stability coefficient should be used as the source data for fitting on the basis of the formulae in line with the processing method in China. Modify Equations (23) and (24) and define $y=\frac{1}{\varphi }$ and $x=\frac{f_{c}\lambda^{2}}{\pi^{2}E_{05}}$ to obtain Equations (27) and (28).

(27) $$y=\frac{1}{a_{c}}x\left(\lambda >\lambda_{p}\right)$$

(28) $$y=\frac{1}{b_{c}}x+1\left(\lambda \leq \lambda_{p}\right)$$

It can be found that the slenderness ratio conversion formula and the stability coefficient conversion formula are essentially a continuous fitting curve. On this basis, the curve can be divided by looking for an appropriate value of $\lambda_{p}$. The characteristic values of the dimension lumber were substituted into the formulae and the stability coefficient values of different grades were calculated in the range of 1 to 200 to slenderness ratio. With $\lambda_{p}=1$ as the step, the source data on both sides of the cut-off point were fitted by the least squares method in the slenderness ratio range of 50~150. According to Equations (23) and (24), the correlation coefficients $a_{c}$, $b_{c}$ and the corresponding error sum of squares for the specimens under different $\lambda_{p}$ were obtained. The error sums of squares of both sides are summed, and the $\lambda_{p}$ value with the minimum error sum of squares was taken as a value of this grade to the correlation coefficients $a_{c}$, $b_{c}$, and $c_{c}$.

The North American Product Standard ANSI/APA PRG 320-2019 classifies laminate materials used in CLT into four mechanical grades and two visual grades. After unifying the treatment method, the elastic modulus and compressive strength standard values in the product standard were substituted into the European standard and the American standard for fitting calculation. The CLT material parameters obtained by fitting are shown in Table 2. After unifying the treatment methods, the CLT material coefficients obtained were not significantly different. In this paper, we took the average values calculated by the European standard and the American standard, and the material coefficients were $a_{c}=1.00$, $b_{c}=2.92$, and $c_{c}=3.89$.

## 4. Materials and Methods

### 4.1. Materials

Japanese larch (*Larix kaempferi*) was harvested from the Dagujia Forest Farm of Liaoning Province, China. The diameters of logs were from 250 to 320 mm. A total of 351 logs with a 4.5 m length was cut into dimension lumber using four-faced sawing; the lumber sizes were 35 mm in thickness and 140 mm in width. The average density and moisture content of the lumber were 0.58 ± 0.07 g/cm^3^ and 12 ± 0.96%, respectively.

After cutting, each lumber was E-rated by nondestructive testing of stress waves. Lumbers with elastic modulus within 11~13 GPa were used as longitudinal laminates and counterparts with 8~10 GPa were used as transverse laminates. The one-component polyurethane was selected as an adhesive, and the pressure was 1.2 MPa. The cross section size of CLT was 300 mm×105 mm and four different lengths were chosen for the research. The lengths of CLT specimens included 3950 mm, 3200 mm, 1950 mm, and 1200 mm and each kind was repeated three times. Relevant information is shown in Table 3.

### 4.2. Compression Test

**Figure 4:**
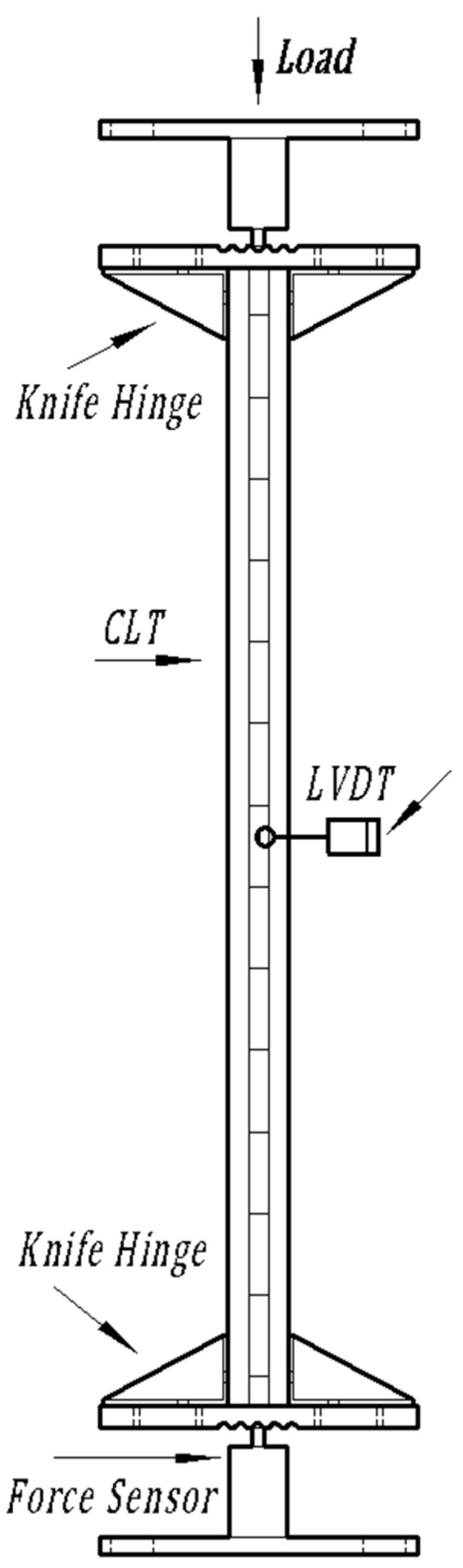
Specimen testing diagram.

The test process was carried out with reference to the standard GB/T 50329-2012, Standard for test methods of timber structures [25]. The JSF-III high-precision static servo-hydraulic control testing machine produced by Chengdu Servo Hydraulic Equipment Co. Ltd. in Chengdu, China was used in the test with the power of 3 KW, dynamic accuracy of ±2%, and static accuracy of ±0.5%. The specimen was supported by a two-way knife hinge, and the specimen was fixed at both ends with the aid of a fixture to avoid horizontal displacement of the end. The diagram of test machine was shown in Figure 4. The test support device meets the five requirements of a two-way knife hinge in GB/T 50329-2012 as an axial compression bar support device. During the test, one pull wire displacement transducer (LVDT) was placed on each side of the span area of the specimen to measure the middle deflection of the specimen.

In the process of specimen loading, it is necessary to first apply a preload $F_{0}$ to the specimen to eliminate the gap between the ends of the specimen and the fixture. In this study, the preloaded load value $F_{0}$ was taken as 10% of the estimated ultimate load value of the specimen. Then, we started to load at 1 kN/s evenly. Since specimens in group C1, C2 were relatively long and thin, the loading later changed to displacement control loading, the speed was 10 mm/min, and when the lateral deflection reached 60 mm, the loading was stopped; C3, C4 group specimens were loaded with 1 kN/s evenly, loading later changed to displacement control loading with the speed of 10 mm/min. The load continued increasing after the specimen load reached the maximum, and stopped loading after the load showed a downward trend with the deflection increasing and dropped to about 80% of the limit load.

After the test, a small specimen of length of 280 mm was cut from each end of each specimen in the C1~C4 group without surface damage to measure the compressive strength along the grain of CLT. The ratio between the stable bearing capacity of the specimen and the compressive strength along the grain of the small specimen is regarded as the stability coefficient of the CLT.

## 5. Results and Discussion

### 5.1. Results and Analysis of Stability Test

**Figure 5:**
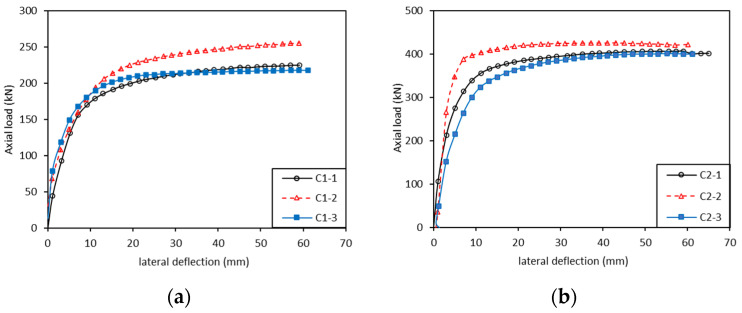

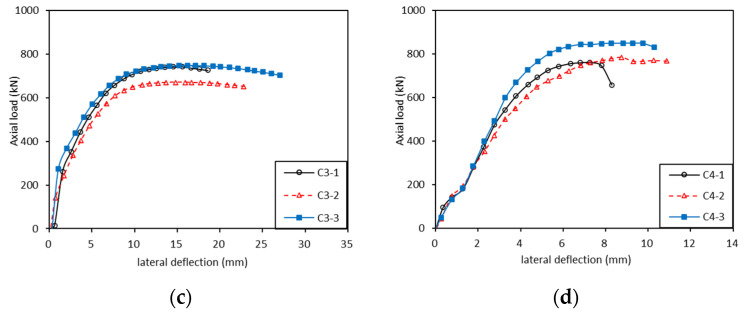
Axial load–lateral deflection curves of CLT specimens in axial compression. (**a**) C1 (3950 mm), (**b**) C2 (3200 mm), (**c**) C3 (1950 mm), (**d**) C4 (1200 mm).

The axial load–lateral deflection curve showed that all specimens in the test were destabilized (Figure 5). C1 and C2 groups of specimens were elastically destabilized, and the surface of the specimen was restored to its original state after the end of axial loading; C3 and C4 groups of specimens were elastoplastically destabilized, and the surface of the specimen was damaged after the end of loading.

The growth trend of lateral deflection is relatively stable with the increase in loads in the C1~C2 groups (Figure 5). In group C1, the load and deflection were approximately proportional when the specimen was in the load range of 0~200 kN. Without significant cracking or wrinkling, the lateral deflection increased slowly with load. After the lateral deflection exceeded 20 mm, the axial load almost stopped growing and the specimen started to bend rapidly. The central part of the axial compression specimen arched obviously, until the lateral deflection reached 60 mm, and the specimen stopped loading. Regarding group C2, the phenomenon was similar to C1 at first, but the proportional range extended to 0~300 kN. When the load reached approximately 60% of the ultimate load (245 kN), specimens started to bend. The instability process and the instability mode of specimens in group C2 were similar to specimens in group C1 and the destructive process of other specimens in groups were similar to the content described.

Observation of groups C3~C4 showed that plastic deformation begins to occur when compared with C1~C2 groups and the ultimate loads were obviously larger than them. In group C3, the first proportional stage occurred in the range of 0~250 kN. The lateral deflection started to grow rapidly from 250 to 600 kN in which the sound of splitting was heard. After the rapid growth, the load reached the limit of about 700 kN and the control mode changed to displacement control loading. With the increase in bending deformation, the loading value gradually decreased. Elastic instability is still the main kind of instability mode that took place in C3 group. Regarding group C4, plastic instability significantly occurred and bending did not occur significantly during the whole process, and other phenomena were similar to C3 group. At the same time, the loads produced shear forces on the cross section, making the transverse layer subjected to rolling shear. The rolling shear strength of a wood product is very low, so the rolling shear failure occurs mainly in the transverse layer. When the slenderness ratio is relatively small (C3~C4), members are closer to strength failure, mainly influenced by shear strength. The fitting curve between axial load and lateral deflection and the trend of stability coefficient with slenderness ratio was similar to the research results of Fu et al. [26] and Chen et al. [27] on the stability performance of slender concrete-filled steel tube columns and bamboo columns.

### 5.2. Stability Coefficient Comparison between Theoretical Curve and Actual Test Results

The test results of the CLT axial compression test are shown in Table 4.

As shown in Table 4, the compressive strength decreased with the increase in slenderness ratio, which was similar to the research conclusions of Wei et al. [28] and Pan et al. [29] on flattened bamboo–wood composite cross-laminated timber and the slender reinforced concrete columns wrapped with FRP, respectively.

**Figure 6:**
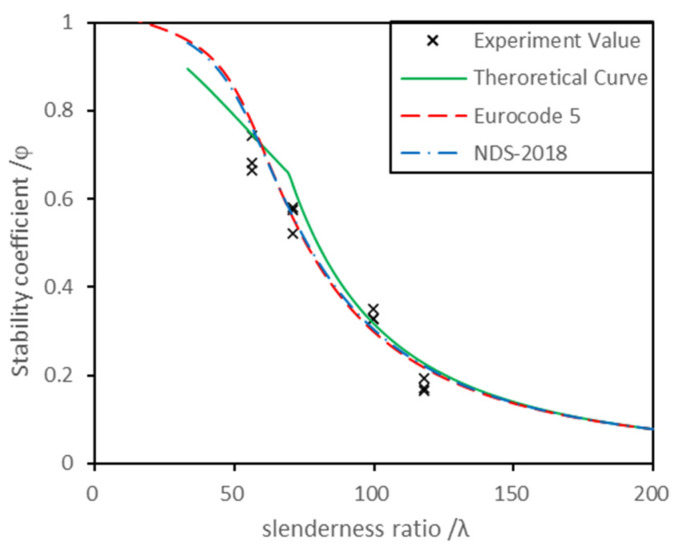
Comparison diagram of the theoretical calculation and experimental results.

By substituting the characteristic values of compressive strength along the grain, the standard values of elastic modulus and the CLT material parameters into the Chinese standard suggested formulae, the fitting curve of the theoretical stability coefficient were obtained. At the same time, the standard values of compressive strength and elastic modulus along the grain were substituted into the European standard and the American standard and the stability coefficient fitting curves were obtained. The measured scattering points of the stability coefficient of each CLT specimen were substituted into the fitting curve, which is shown in Figure 6. The stability coefficient curves fitted with the scattering points well. The overall average deviation was 8.15%, and the maximum average deviation for each group was 15.42% (C1). The maximum average deviation occurred at the maximum slenderness ratio. With an increase in the slenderness ratio, the stability coefficient gradually decreased, and the relative deviation became larger. In addition, the stability bearing capacity of the members with large slenderness ratio was easily affected by the initial geometric defects of the members.

The average deviation of the stability coefficient calculated following the suggested formulae from the European standard is 5.43% and that from the American standard is 3.73%. The maximal deviation with the European standard and American standard is 12.32% and 11.70%, respectively. They both occurred at the critical slenderness ratio (λ=70), which should be on account of the discontinuity of the Chinese suggested formulae. The average deviation between the Chinese standard and American standard was lower than that of the European standard. This may be because the American standard slenderness ratio calculation method used in this paper takes the shear deformation factor into account, which is similar to the treatment method in the Chinese standard suggested formulae.

The resulting critical slenderness ratio of this paper was calculated to be about 70. Theoretically, the C1 (*λ* = 118.07) and C2 (*λ* = 99.49) groups showed elastic instability while the C3 (*λ* = 70.86) and C4 (*λ* = 56.27) groups showed elastic–plastic instability. In the actual test process, groups C1 and C2 had complete elastic instability, and group C4 had complete elastoplastic instability. However, group C3 still had a small amount of elastoplastic instability, which may because the slenderness ratio of group C3 was close to the critical slenderness ratio. The experimental results basically meet the theoretical inference.

## 6. Conclusions

In this study, CLT compression members with four slenderness ratios were used to test the stability coefficients and CLT stability coefficient formulae modified from the GLT stability coefficient formulae in the Chinese standard were used to calculate the theoretical values of these members and then compared with test results. The main conclusions are as follows:

(1) The experiment showed that with the increase in slenderness ratio of the CLT axial compression member, the stability bearing capacity gradually decreased. The C1 (*λ* = 118.07) and C2 (*λ* = 99.49) specimens were mainly characterized by elastic instability, and there was almost no damage to the specimens during the test; the C4 (*λ* = 56.27) specimens mainly showed elastic–plastic instability, and folding and shear failure occurred on the surface of the specimen. Both instability phenomena occurred in group C3 (*λ* = 70.86).
(2) By considering the CLT as a split limb, the longitudinal lamina as the column limb, and the transverse lamina as the connection between the component limbs, the slenderness ratio conversion theory, was proposed. Combined with the γ coefficient method, the calculation method of the stability coefficient for CLT elements were proposed.
(3) The material parameters $a_{c}$, $b_{c}$, $c_{c}$ of the stability coefficient calculation formulae were 1.00, 2.92, and 3.89, respectively. The curve fit well with the calculated results of the European standard and American standard, and the average deviation was less than 6%. The average deviation between the fitting curve and the actual test results was 8.15%.

## Figures and Tables

**Table 1:** The process modes of parameters in the stability coefficient formulae in different countries.

Countries	Loading Duration	Resistance Partial Coefficient	Form of Formula
China	$K_{cr,DOL}=K_{DOL}$	$\gamma_{cr,R}=\gamma_{R}$	segmented
Europe	$K_{cr,DOL}=K_{DOL}$	$\gamma_{cr,R}=\gamma_{R}$	continuous
Canada	$K_{cr,DOL}=1.0$	$\gamma_{cr,R}\neq \gamma_{R}$	continuous
America	$K_{cr,DOL}=1.0$	$\gamma_{cr,R}\neq \gamma_{R}$	continuous

Notes. $K_{cr,DOL}$ is the coefficient of load duration’s influence on stable bearing capacity; $K_{DOL}$ is the coefficient of load duration’s influence on the strength of lumber or wood products; $\gamma_{cr,R}$ is the resistance partial coefficient of the stability bearing capacity meeting the reliability requirements; and $\gamma_{R}$ is the resistance partial coefficient to meet reliability requirements.

**Table 2:** CLT material coefficients based on different standards.

Coefficient	PRG 320		Average
	Eurocode: 5	NDS-2018	
$a_{c}$	0.99	1.02	1.00
$b_{c}$	3.06	2.77	2.92
$c_{c}$	3.81	3.98	3.89

**Table 3:** CLT specimen materials.

Number	Repetition Times	Size/mm	Slenderness Ratio
C1	3	3950 × 300 × 105	118.07
C2	3	3200 × 300 × 105	99.49
C3	3	1950 × 300 × 105	70.86
C4	3	1200 × 300 × 105	56.27

**Table 4:** Results of CLT axial compression experiment.

Groups	Slenderness Ratio	$F_{s}/kN$	$V_{s}/\%$	$F_{l}/kN$	$V_{l}/\%$	$\varphi_{t}$	$\varphi_{c}$
C1	118.07	252.00	6.33	1311	4.42	0.192	0.227
C2	99.49	408.67	3.00	1219	7.34	0.335	0.320
C3	70.86	719.33	4.87	1286	3.90	0.559	0.630
C4	56.27	796.70	4.84	1143	9.00	0.697	0.745

Notes. $F_{s}$ is the stable bearing capacity of the CLT specimen; $V_{s}$ is the coefficient of variation of the test stable bearing capacity; $F_{l}$ is the CLT ultimate bearing capacity, taking the minimum value of bearing capacity of the same group of specimens; $V_{l}$ is the coefficient of variation of the bearing capacity of the specimen; $\varphi_{t}$ is the actual test stability coefficient of the specimen, taking the ratio of the stability bearing capacity to the ultimate bearing capacity; and $\varphi_{c}$ is the theoretical calculation stability coefficient.

## Data Availability

Data used in this study are included in this article.
